# Elevated expression of the *IGF2* mRNA binding protein 2 (IGF2BP2/IMP2) is linked to short survival and metastasis in esophageal adenocarcinoma

**DOI:** 10.18632/oncotarget.10439

**Published:** 2016-07-06

**Authors:** Ahmad Barghash, Nicole Golob-Schwarzl, Volkhard Helms, Johannes Haybaeck, Sonja M. Kessler

**Affiliations:** ^1^ Center for Bioinformatics, Saarland University, Saarbruecken, Germany; ^2^ School of Computer Engineering and Information Technology, German Jordanian University, Amman, Jordan; ^3^ Institute of Pathology, Medical University of Graz, Austria; ^4^ Department of Pharmacy, Pharmaceutical Biology, Saarland University, Saarbruecken, Germany

**Keywords:** IGF2BP2/IMP2/p62, barret's esophagus, esophageal squamous carcinoma, esophageal adenocarcinoma, metastasis

## Abstract

Esophageal adenocarcinoma (EAC) represents the sixth leading cause of cancer-related deaths and develops in Barret's esophagus affected tissues. The *IGF2* mRNA binding protein IMP2/IGF2BP2/p62 was originally identified as an autoantigen in hepatocellular carcinoma. Aim of this study was to investigate the expression and prognostic role of IMP2 in EAC. Human EAC and Barret's esophagus tissue showed overexpression of IMP2, particularly in tumors of increased size and in metastatic tissues. Molecular classification based on published gene signatures of esophageal cancer revealed a specific subtype, in which the expression of *IMP2* is high. According to GO and KEGG pathway analyses, genes showing highly correlated expression with *IMP2* are associated with growth, proliferation, metabolism, inflammation, and cancerous processes. Clustering of EAC samples according to published survival marker genes strongly suggests that *IMP2* overexpressing samples show poor survival. Finally, IMP2 expression correlated with short survival in patients with EAC or esophageal squamous carcinoma. Our data indicate that IMP2 might be a useful prognostic marker for Barret's esophagus and EAC.

## INTRODUCTION

Esophageal cancer represents the eighth most common malignancy and the sixth most common cause of cancer-related deaths worldwide. Most cases of esophageal cancers are either esophageal adenocarcinoma (EAC) or squamous cell carcinoma (ESCC). ESCC dominates in Asian countries and EAC in Western countries [1]. EAC is the cancer with the fastest increasing incidence showing a 6-fold increase in the past decades. Esophageal adenocarcinoma has a poor overall 5-year survival rate due to presentation with an advanced disease stage, in which treatment is ineffective. Barrett's esophagus is an established precursor of EAC, in which the squamous epithelium of the esophagus is affected by metaplastic changes. Patients with Barrett's esophagus have a 30– to 60-fold increased risk of EAC development [2]. Invasive cancer will develop in almost 50% of patients with high-grade dysplasia who do not undergo esophageal resection [3]. However, in clinical practice diagnosis of the high-risk precancerous lesion Barret's esophagus and detection of the transition to neoplasia is difficult and needs to be improved [4].

Recently, autoantibodies against the insulin-like growth factor 2 mRNA binding protein (IMP) IMP2/p62, which was originally identified as an autoantigen in a hepatocellular carcinoma patient [5], were shown to be elevated in patients with esophageal squamous carcinoma [6].

Aim of this study was to investigate the role of IMP2 expression in Barret's esophagus and esophageal adenocarcinoma and to test its prognostic relevance.

## RESULTS

### IMP2 is overexpressed in Barret's esophagus and esophageal adenocarcinoma

**Figure 1:**
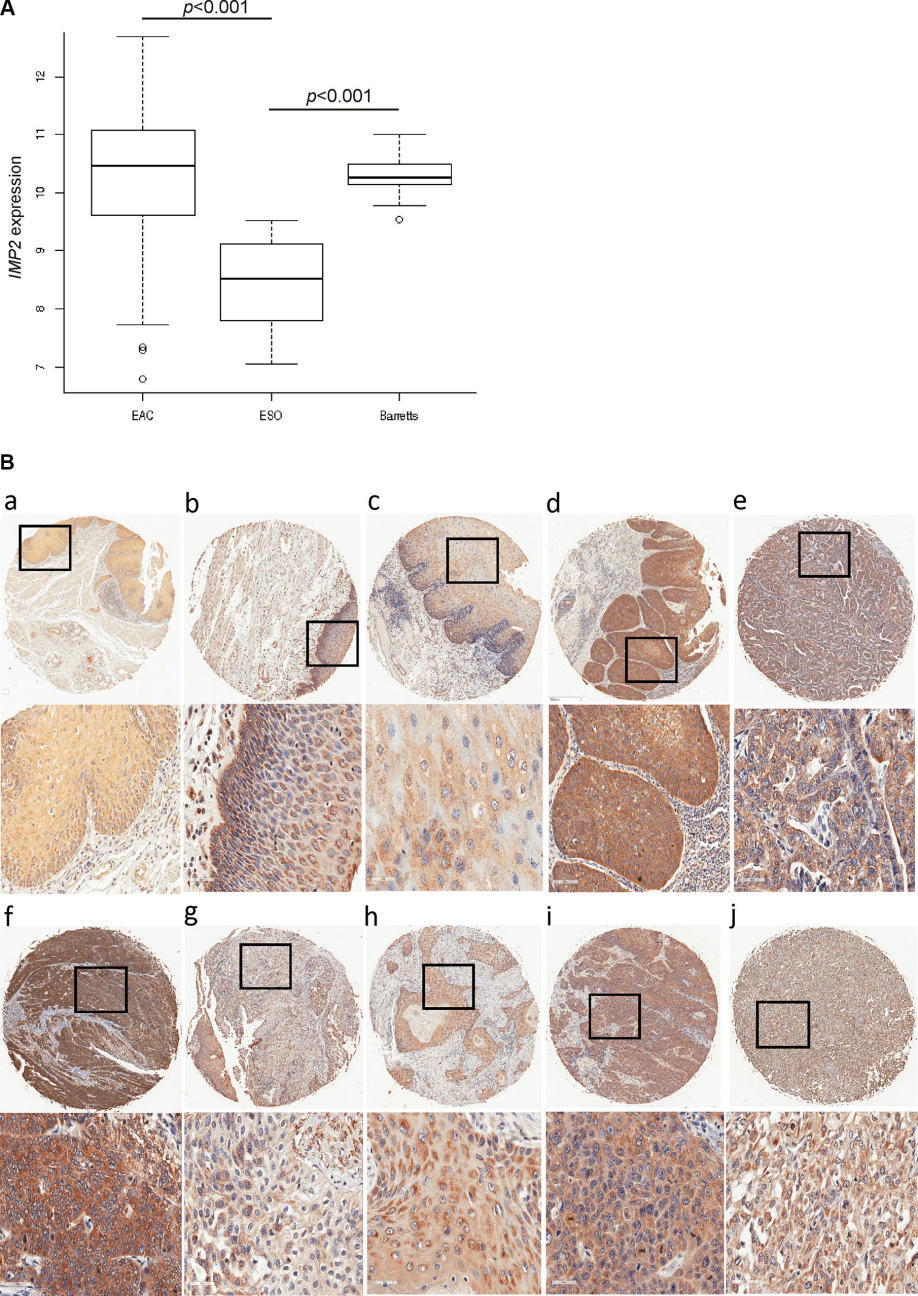
IMP2 overexpression in esophageal hyperplasia and cancer (**A**) Expression analysis of *IMP2* in Barret's esophagus (Barrets) (*n* = 15), esophageal adenocarcinoma (EAC) (*n* = 64), and normal esophagus (*n* = 28) (ESO) samples (GSE13898). Error bars show the interquartile range. (**B**) Representative immunohistochemical staining for IMP2 in non-malignant normal esophagus (a), chronic esophagitis (b), mild atypical and severe hyperplasia (c, d), esophageal adenocarcinoma grade 2 and 3 (e, f), adenosquamous carcinoma (g), and esophageal squamous cell carcinoma grade 1, 2, and 3 (h, i, j). Scale bars: 50 μm.

We investigated *IMP2* expression in a large patient cohort (GSE13898) of more than 60 esophageal adenocarcinoma cases. *IMP2* was distinctly overexpressed in tumor (Figure 1A) compared to normal tissue. Interestingly, *IMP2* was also overexpressed in tissues of the precancerous lesions Barret's esophagus including low grade and high grade dysplasia tissues (Figure 1A). Barret's esophagus tissues without dysplasia did not show an altered expression of *IMP2* compared to normal squamous epithelium (GSE28302; data not shown). In order to confirm these results on protein level, immunohistochemical staining against IMP2 was performed on a tissue microarray (Table 1). In fact, although IMP2 could be detected in normal esophageal epithelium, expression was distinctly increased in esophageal squamous carcinoma tissues and adenocarcinomas tissues compared to normal and esophagitis tissues (Figure 1B; Table 1; *p* = 0.0103). In tissues showing severe hyperplasia, increased *IMP2* gene expression levels were observed (Figure 1B) compared to mild atypical hyperplasia (Table 1; *p* = 0.027) and to normal esophageal tissue (Table 1; *p* = 0.047).

**Table 1:** Esophageal tissue microarray

	Intensity of IMP2 immunohistochemical staining				*p*-value
	0	1	2	3	
gender					
female	0	3	7	5	
male	1	18	23	15	
	
age					
mean	56 +/−0	55.7 +/−2.3	54.2 +/−2.3	58.7 +/−1.5	
	
normal esophageal tissue	0	0	5	0	
cancer adjacent tissue	0	3	2	0	
chronic esophagitis	0	3	5	0	
mild atypical hyperplasia	0	5	2	0	
moderate and severe atypical hyperplasia	0	0	0	2	^a^0.0027^b^0.047
adenocarcinoma	0	4	6	6	^c^0.0103
adenosquamous carcinoma	0	0	1	0	
squamous carcinoma	1	5	9	5	
metastatic adenocarcinoma	0	0	0	3	^d^0.0042
metastatic squamous carcinoma	0	1	0	4	

Table displays details of esophageal tissues referred to the intensity of IMP2 immunohistochemical staining (score 0 = no staining, score 1 = low intensity, score 2 = medium intensity, score 3 = strong intensity).

a compared to mild atypical hyperplasia;

b compared to normal esophageal tissue;

c compared to normal esophageal and cancer adjacent tissue;

b compared to adenocarcinoma, adenosquamous carcinoma, and squamous carcinoma.

### Molecular classification of *IMP2* overexpressing samples

**Figure 2:**
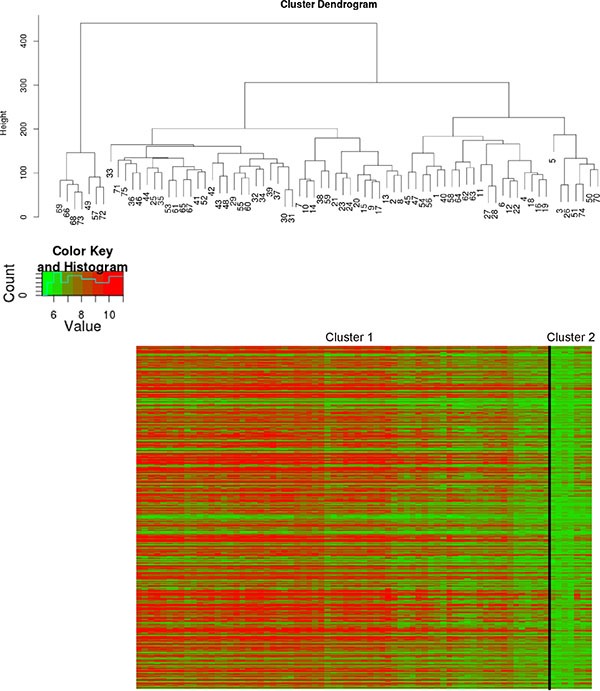
Molecular classification of IMP2 overexpressing samples Hierarchical clustering of esophageal adenocarcinomas (*n* = 64; GSE13898) according to Greenawalt's Cluster C marker genes [7]. Cluster dendogram (upper panel) and heatmap with Cluster C marker genes grouped in the two clusters cluster 1 and cluster 2 (lower panel) are shown.

Since molecular subclasses of esophageal cancer have been described in the literature [7], the samples of GSE13898 were clustered according to the marker genes of Greenawalt's Cluster C [7] being specific for esophageal cancer. Hierarchical clustering revealed two main clusters in the gene expression dataset GSE13898 (Figure 2). SNR analysis revealed that *IMP2* as well as 93% of Greenawalt's Cluster C marker genes belong to the large Cluster 1 confirming that *IMP2* overexpression was associated with the gene expression profile of Greenawalt's Cluster C (Supplementary Table S1). Furthermore, analysis of GO functional annotations showed that genes having strongly correlated expression with *IMP2* (threshold 0.85) showed enrichment for processes stimulating growth (Supplementary Table S2). KEGG pathway analysis confirmed an involvement of these genes in signaling pathways, such as MAPK and Jak-STAT pathway, both known to be activated during proliferation and carcinogenesis, as well as in pathways related to metabolism, inflammation, post-translational modifications, protein-processing, and cancer (Supplementary Table S3).

### *IMP2* expression correlates with tumor size, clinical stage, metastasis, and short survival

**Figure 3:**
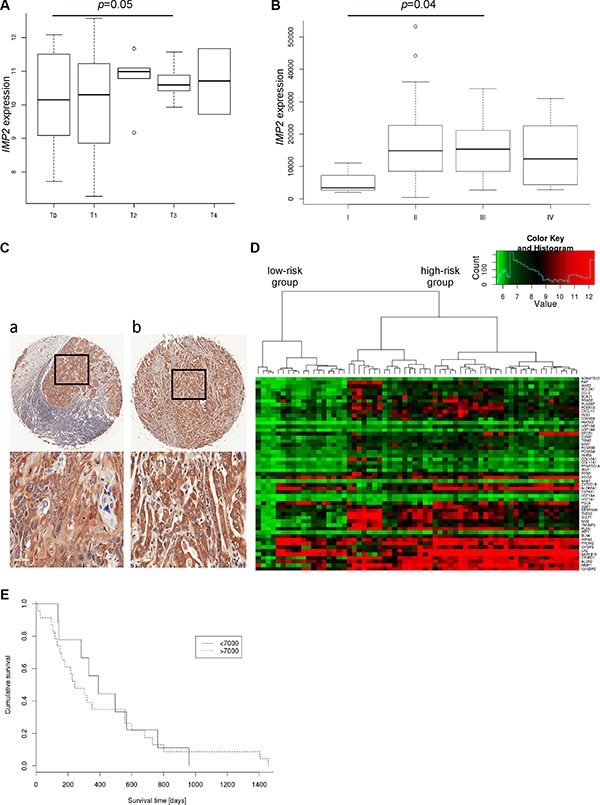
IMP2 expression increases with tumor size, metastasis and poor prognosis (**A**) Expression of *IMP2* in esophageal adenocarcinoma (*n* = 64) grouped by tumor stage (T0–T4; T0: *n* = 13, T1: *n* = 9, T2: 6, T3: *n* = 15, T4: *n* = 2) (GSE13898). Error bars show the interquartile range. (**B**) Expression of *IMP2* in esophageal adenocarcinoma (*n* = 20) and esophageal squamous carcinoma (*n* = 44) grouped by clinical tumor stages. (**C**) Representative immunohistochemical staining for IMP2 in metastatic esophageal squamous cell carcinoma (a) and adenocarcinoma (b). Scale bars: 50 μm. (**D**) Heatmap showing expression of IMP2 (bottom row) and 52 marker genes for poor prognosis described by Pennathur et al. [8] in esophageal adenocarcinoma (*n* = 64; GSE13898). (**E**) Kaplan-Meier survival plot referring to low and high *IMP2* expression levels in TCGA dataset (*n* = 57). High expression are those samples with *IMP2* expression higher than 7000. Low expression < 7000, respectively.

*IMP2* expression was increased with tumor size (Figure 3A) and clinical tumor stage (Figure 3B). Although, *IMP2* expression was not related to lymph node metastasis (data not shown), distant metastasis showed increased IMP2 protein levels compared to primary tumor tissue (Figure 3C; Table 1; *p* = 0.0042). In order to test whether *IMP2* expression correlates with prognosis, we analyzed the marker genes shown to be predictive for survival defined by Pennathur et al. [8] in the gene expression dataset GSE13898. Pennathur's marker genes had similar expression profiles in this dataset suggesting similar survival relations for the clustered samples. Therefore, gene expression signature strongly suggests that *IMP2* overexpressing samples correspond to Pennathur's high-risk group (Figure 3D). Finally, Kaplan-Meier plot of TCGA samples confirmed that high *IMP2* expression is linked to a shorter survival time in esophageal cancer patients (Figure 3E; *p* = 0.008).

## DISCUSSION

IMP2/p62 was originally identified as a tumor-associated auto-antigen with auto-antibodies against p62 detected in HCC patients [5]. Autoantibodies against IMP2 have recently also been described to be elevated in ESCC [6, 9]. Our analysis of two large human esophageal cancer cohorts with about 60 tumor samples for gene expression and 50 tumor samples for protein expression showed strongly increased expression of *IMP2* in the majority of esophageal cancer patients. These data are supported by another study reporting elevated levels of IMP2 in esophageal cancer tissue in a rather small patient cohort, in which the specific esophageal cancer type was not defined [10].

In clinical practice, diagnosis of the high-risk precancerous lesion Barret's esophagus as well as the transition to neoplasia is rather difficult and its accuracy needs improvement [4]. IMP2 might serve as a useful biomarker to detect high-risk lesions since other suggested biomarkers of Barrett's esophagus progression were not able to detect dysplasia at predictive accuracy [11].

Molecular profiles, such as gene signatures, could be used for an individualized therapy depending on the pathways activated or inactivated in the tumor tissue. Only few studies describe gene profiles for esophageal cancer so far. *IMP2* overexpressing EAC samples were related by us to the published Cluster C, which has been shown to be specific for EAC [7]. This cluster includes the previously published SPARC and proliferation clusters [12, 13]. *SPARC* expression itself was shown to correlate with poor survival in esophageal cancer [14]. Based on both published marker genes that are predictive for survival [8] and on a Kaplan-Meier survival analysis we showed that high *IMP2* expression was linked to short survival. The prognostic relevance of *IMP2* expression has been described for other cancer types [15–17].

*IMP2* expression in GSE13898 was correlated to overexpression of genes involved in metabolism. This is in line with other studies which reported that IMP2 is involved in obesity and liver steatosis [18–20]. In steatohepatitis IMP2 overexpression led to the accumulation of free cholesterol and the activation of a fatty acid elongase [21, 22]. The observed correlation of *IMP2* and signaling pathways such as MAPK and Jak-STAT seems reasonable since IMP2 expression results in increased levels of *IGF2* [20], which can activate both of these pathways. A link between inflammation and IMP2 expression was previously shown. The observed relationship between IMP2 and genes regulating post-translational modifications and protein-processing is more likely to be due to co-expression of genes regulating IMP2 activity. The level of activity of mRNA-binding proteins depends on their post-translational modifications [23].

Taken together, our data show that IMP2 might serve as both a diagnostic and prognostic marker for esophageal cancer.

## MATERIALS AND METHODS

### Tissue microarray and immunohistochemistry

Esophageal carcinoma tissue microarray was purchased from US Biomaxx (#ES804, Rockville, United States). Details of esophageal tissues are given in Table 1. Of the total 80 cases, eight cases did not contain the respective tissue on the slide and thus could not be analyzed. Immunohistochemical stainings against IMP2 were performed as previously described [15] using the Dako Envision DAB Kit (#K4003, Dako, Germany) for antibody detection according to the manufactor's instructions.

### Statistical analysis

Data analysis and statistics of experimental data were performed using either R software or Origin software (OriginPro 8.1G; OriginLabs). Differential expression analysis was based on the Kolmogorov–Smirnov test. Fisher-exact -test was used for categorical data. Pearson correlation was applied to detect correlations between genes of interest. All tests are two-sided and differences were considered statistically significant when *p* values were less than 0.05.

### Analysis of human Gene Omnibus (GEO) datasets

Preprocessed and normalized data from the DNA microarray (Illumina human V2) GEO dataset (GSE13898 [14]) was analyzed for differential gene expression between esophageal adenocarcinoma (*n* = 64), Barret's esophagus (*n* = 15) including no grade (*n* = 2), low grade (*n* = 7), and high grade dysplasia (*n* = 6), and non-tumor tissues (*n* = 28) samples. GSE28302 [24] was analyzed for differential gene expression between normal esophageal squamous tissue (*n* = 9) and Barret's esophagus without dysplasia (*n* = 22). Pearson correlation was applied to detect possible co-expressions between genes of interest and other genes in the dataset. For sets of co-expressed genes, enriched Gene Ontology terms were identified from the biological processes (BP) track using Bioconductor package GOSim [25]. Participation of co-expressed genes in the same KEGG pathway was tested using Bioconductor package org.HS.eg.

For the same GEO dataset GSE13898, unsupervised hierarchical clustering of the expression levels of IMP2 and the 93 marker genes forming the esophageal cancer cluster “C” in [7] was performed. For each marker gene, the signal-noise-ratio (SNR) was calculated as previously described [26] to test the stability of the suggested clustering.

To get a hint about possible survival relations, SNR values for 53 marker genes constructing a risk classifier provided in Pennathur et al. [8] were computed. By unsupervised hierarchical clustering, these authors showed that 59 suggested marker genes divide samples of 64 patients into 2 well-differentiated clusters, in which patient samples show a different survival profile. In the GSE13898 dataset, 53 out of the 59 provided marker genes were present. Similarly, unsupervised hierarchical clustering was applied to the samples of untreated patients with esophageal adenocarcinoma (EAC) using available marker genes in addition to IMP2.

### Analysis of human The Cancer Genome Atlas (TCGA) dataset

Level 3 RNA-Seq data and related clinical datasets were obtained from TCGA (downloaded on April 22, 2016). Datasets were analyzed in R-cran environment using Bioconductor package edgeR [27–29] for differential gene expression between different clinical tumor stages. Information about clinical tumor stage was available for *n* = 64 samples. Samples of TCGA dataset informative for survival time (*n* = 57) were used for survival analysis by Kaplan-Meier survival plot.

## SUPPLEMENTARY MATERIALS TABLES








