# Supplementary material for: Elevated expression of the IGF2 mRNA binding protein 2 (IGF2BP2/IMP2) is linked to short survival and metastasis in esophageal adenocarcinoma
Source: Oncotarget. 2016 Jul 6;7(31):49743–50. doi: 10.18632/oncotarget.10439 (PMC5226544; doi:10.18632/oncotarget.10439)
Supplement: Supplementary file 1 [file oncotarget-07-49743-s001.pdf]

Elevated expression of the *IGF2* mRNA binding protein 2 (IGF2BP2/IMP2) is linked to short survival and metastasis in esophageal adenocarcinoma

Supplementary Materials

Supplementary Table S1: SNR analysis of cluster C marker genes. See Supplementary_Table_S1

Supplementary Table S2: Enriched go terms. See Supplementary_Table_S2

Supplementary Table S3: Gene pathways. See Supplementary_Table_S3
